# Young Women’s Attitudes and Behaviors in Treatment and Prevention of UTIs: Are Biomedical Students at an Advantage?

**DOI:** 10.3390/antibiotics12071107

**Published:** 2023-06-26

**Authors:** Ivan Jerkovic, Josipa Bukic, Dario Leskur, Ana Seselja Perisin, Doris Rusic, Josko Bozic, Tomislav Zuvela, Sara Vuko, Jonatan Vukovic, Darko Modun

**Affiliations:** 1 Department of Internal Medicine, University Hospital of Split, Spinciceva 1, 21000 Split, Croatia; ijerkovic@kbsplit.hr (I.J.); jvukovic@mefst.hr (J.V.); 2 Department of Pharmacy, University of Split School of Medicine, Soltanska 2, 21000 Split, Croatia; jbukic@mefst.hr (J.B.); dleskur@mefst.hr (D.L.); aperisin@mefst.hr (A.S.P.); tomislav.zuvela@mefst.hr (T.Z.); sara.vuko@mefst.hr (S.V.); dmodun@mefst.hr (D.M.); 3 Department of Pathophysiology, University of Split School of Medicine, Soltanska 2, 21000 Split, Croatia; jbozic@mefst.hr

**Keywords:** UTIs, women, treatment, prevention

## Abstract

We wanted to investigate whether students who study within biomedical fields (i.e., medicine, pharmacy science) differ from those whose studies are not connected to the biomedical field in terms of their attitudes and behaviors related to urinary tract infections (UTIs). This was a cross-sectional survey-based study conducted among 392 female students, of whom 243 attended a biomedical school and 149 (38.0%) attended a non-biomedical school, using a previously published tool. The survey was distributed as an online link via student representatives at different faculties. Only 22 (5.6%) of women felt that they could not recognize a UTI. A greater proportion of biomedical students wiped front to back, while significantly more non-biomedical students chose cotton underwear and avoided daily sanitary pads compared to biomedical students. As many as 215 (54.8%) women stated that they used cranberry preparations. Biomedical students showed greater awareness about possible resistance to repeated treatment (*p* = 0.002) and greater knowledge of possible interactions of antibiotics (*p* < 0.001). This study reveals that young women are confident in recognizing an UTIs, are open to alternative treatments, and would consider UTI management in a pharmacy setting. However, it reveals that there might be gaps in their knowledge regarding antibiotic resistance risks, possible interactions, and efficacy of available preparations, as participants from the group of biomedical students showed greater knowledge and different behaviors.

## 1. Introduction

Urinary tract infections (UTIs) are among the most commonly diagnosed outpatient infections [1]. Although uncomplicated UTIs may be self-limited in around 50% of women, data indicate that antibiotics are prescribed to three-quarters of symptomatic women [2]. Accordingly, while professional societies have developed recommendations for treatment, practice patterns vary widely, showing discordance between treatment guidelines and clinical practice [1]. Research indicates that two thirds of prescriptions are not in line with established guidelines; for example, research has shown that compliance with guidelines may vary from around 60% to up to 91% in the Military Health System [1,2]. Antibiotic prescription rates for UTIs vary between 59% and 95% around Europe, while prescribing according to the guidelines is reported to range from 23.8% to 66.7% [3,4,5].

When prescribing treatments, physicians may take various factors into consideration that may affect their final decision that may not be fully aligned with treatment guidelines. These may include patient preferences, socio-demographic background, how many UTIs the patient has had, other comorbidities, physician preference and other factors. However, the published research is inconclusive. A study by van Driel et al. has suggested that the presence of environmental and socio-demographic risk factors for antibiotic resistance does not influence the empiric choice of antibiotics in women with uncomplicated UTI [6,7]. Different interventions have been developed to improve general practitioners’ (GPs) UTI care. Different stewardship interventions, such as electronic order set and audit and feedback, can be implemented to improve physicians’ adherence to treatment guidelines and improve GPs treatment for patients with UTI. Other stewardship interventions may include reflex urine cultures, computerized decision support systems, and modified reporting of urine culture results [7,8]. A systematic review of such interventions concluded that they may lead to increase of treatments with first-choice antibiotics and a decrease number of treatments with broad-spectrum antibiotics [2]. For example, at-home testing kits may ease the burden GPs face when treating UTIs, as they may aid the accuracy of UTI diagnosis and help to distinguish patients who would benefit from antibiotic treatment from those who do not need it [9,10]. There have been vaccines developed for prevention of recurrent UTIs as well, offering greater protection than antimicrobial prophylaxis [11].

Several factors beyond the infection itself may affect antibiotic treatment of UTIs, such as history of cervical cancer and differences between sociodemographic groups [12]. UTI will affect up to 60% of women at least once in their lifetime; female sex is one of the main risk factors for the development of UTIs, probably due to anatomical factors [13,14]. Studies have noted greater treatment rates among younger women, explaining this as being due to lack of knowledge in self-treatment as compared to older women, with the result of increasing demands for antibiotic treatment by younger women [12].

However, although patients presenting with more symptoms are more likely to receive an antibiotic [6], a proportion of patients with cystitis do not require antibiotics; as a result, the recent Swedish guidelines (2017) do not recommend antibiotic treatment for uncomplicated cystitis with mild symptoms [12,15].

Although UTIs have low morbidity, they affect different areas of patients’ lives [16,17,18]. Recurrent UTIs can have substantial effects on quality of life, impacting both intimate and social relationships [18]. Patients have reported missing work and social commitments. Furthermore, bathroom stops interfere with childcare and daily routine, while pain reduces overall effectiveness when performing various tasks [16,17]. Considering this impact on quality of life, researchers have concluded that prophylaxis for recurrent UTIs is underutilized, with prescription rates being around 40% for women who have experienced three UTIs per year [18].

According to current medical practice in Croatia, urinary tract infections may be managed without GP consultation through over-the-counter (OTC) medicines and other preparations in women only. Urinary tract infections may adversely affect quality of life, leading to missed work or social engagement; however, certain medications used in self-management of UTIs may alter the pharmacokinetics of other concomitantly used medications and promote antimicrobial resistance, leading to more complicated cases of UTI in the future [19]. A recent study from Pakistan reported resistance to ciprofloxacin in 51.8% of *E. coli* isolates and ceftriaxone resistance in 66.7% of *E. coli* isolates and 33.3% of *K. pneumoniae* in patients with urinary tract infections [20]. As it is expected that a great number of women will experience a urinary tract infection at least once in their lifetime, investigating female students’ attitudes and behaviors related to urinary tract infections and their ability to prevent and recognize UTIs may be of great value to this group. Furthermore, we wanted to investigate whether students who study biomedical fields (i.e., medicine, pharmacy science) differ from those whose studies are not connected to the biomedical field.

## 2. Results

This study included a total of 392 female students enrolled in the academic year 2022/2023 at the University of Split, of whom 243 (62.0%) with median age of 22 years (IQR 20.0–24.7 years) attended a biomedical school and 149 (38.0%) with median age of 24 years (IQR 22.0–29.0 years) attended a non-biomedical school. A little less than half of all students stated they had had at least one UTI in the previous year (172, 43.9%), and there was no difference between the two groups regarding the proportion of students who had at least one UTI in the previous year between the biomedical and non-biomedical group (98, 40.3% vs. 74, 49.7%; *p* = 0.071).

Women reported symptoms of feeling pain or burning when passing urine (250, 63.8%), frequent urination (242, 61.7%), urgent urination of small volumes of urine (droplets) (205, 52.3%), pain or uncomfortable pressure in the lower abdomen (162, 41.3%), scent of urine (101, 25.8%), loin (lower back) pain (54, 13.8%), blood seen in urine (24, 6.1%), and high body temperature (chills, fever) (17, 4.3%) when experiencing an episode of UTI. There was no significant difference among the two groups in confidence of recognizing an UTI (*p* = 0.166). Most women (331, 84.4%) felt they did not need antibiotics for every UTI, with no significant difference between the two groups (211, 86.8% vs. 120, 80.5%; *p* = 0.096). Overall, 267 (68.1%) of women felt that they could recognize a UTI and 103 (26.3%) felt that they could sometimes recognize a UTI, while only 22 (5.6%) felt that they could not recognize an UTI. Prescription medications for prevention of UTI were provided to 30 (7.7%) of women (*p* = 0.535). Antibiotics for recurrent UTI were prescribed to 87 (22.2%) of the women, 56 (23.0%) in the biomedical and 31 (20.8%) in the non-biomedical group, for a future repeated episode of UTI (*p* = 0.605). Of these, 38 (43.7%) stated that they obtained it just in case in order to have it on hand in the future, 34 (39.1%) for holiday upon their request, and 15 (17.2%) in order to always have an antibiotic (*p* = 0.384). Women who provided data on prescribed prophylaxis most often stated that it was nitrofurantoin, while one stated that it was norfloxacin and one a combination of sulfamethoxazole and trimethoprim.

Upon contracting a UTI, roughly one third of women (113, 28.8%) consulted a GP as soon as possible (70, 29.8% vs. 43, 28.9%; *p* = 0.991), while around half (206, 52.6%) increased their liquid intake and then consulted a general practitioner (GP) if this did not diminish their complaint (124, 51.0% vs. 82, 55.0%, *p* = 0.441). A quarter of women (98, 25.0%) increased their liquid intake and used painkillers prior to consulting a GP (61, 25.1% vs. 37, 24.8%; *p* = 0.952), and around 15% (*n* = 57) stated that they just waited a few days prior to consulting a GP (30, 12.3% vs. 27, 18.1%; *p* = 0.116). Less than 5% (*n* = 17) stated that they self-medicated with leftover antimicrobials (13, 5.3% vs. 4, 2.7%, *p* = 0.307), while significantly more non-biomedical students used dietary supplements for a UTI (19, 7.9% vs. 24, 16.1%, *p* = 0.011).

Different behaviors of women when they have an UTI are shown in Table 1. A greater proportion of biomedical students wiped front to back, while significantly more non-biomedical students chose cotton underwear and avoided daily sanitary pads compared to biomedical students (Table 1). As a preventative measure for UTI, increasing fluid intake was most frequent, followed by not postponing micturition and using cotton underwear. Complete emptying of the bladder was a measure taken by 55.9% of women while they had UTI and by 41.6% for prevention of UTI (Table 1). To completely empty the bladder, most women relaxed the bladder (172, 43.9%), while a proportion of them strained when urinating (67, 17.1%), urinated for a long time (61, 15.6%), or sat up straight with feet on the ground (50, 12.8%), and less than 10% tilted the pelvis when urinating stopped (36, 9.2%).

As many as 215 (54.8%) women stated that they used cranberry preparations, of whom 35 (16.3%) used it for prevention of UTIs and 180 (83.7%) during a UTI. Vitamin C was a choice for 125 (31.9%) women, with 47 (37.6%) using it during a UTI and 78 (62.4%) using it every day for prevention of UTIs. Oral probiotics were an option for 123 (31.4%) women, with 76 (61.8%) using them during a UTI and 47 (38.2%) using them every day for prevention. D-mannose was used by 97 (24.7%) women, of whom 16 (16.5%) stated they used it for prevention and 81 (83.5%) that they used it during a UTI. Vaginal probiotics were used by 90 (23.0%) women, 78 (86.7%) of whom used them during a UTI and 12 (13.3%) of whom stated that they used them for preventive purposes. Preparations of *Arctostaphylos uva-ursi* were used by 66 (16.8%) women, most of whom used them during a UTI (62, 93.9%), while two other traditional herbal medicines were used by 51 (13.0%) and 53 (13.5%) women, mostly during UTIs as well (46, 90.2% and 48, 90.6%). There was no significant difference in the purpose of use of the mentioned medicines and supplements between the two studied groups (Table 2).

Biomedical students showed greater awareness about possible resistance to repeated treatment (*p* = 0.002) as well as greater knowledge about possible interactions between antibiotics (*p* < 0.001, Table 3). A greater proportion of them felt that it was safe to postpone antibiotic treatment compared to non-biomedical students (*p* = 0.036, Table 3). Roughly half of the students agreed or fully agreed with statements that vaginal probiotics can either help to reduce symptoms of infection or help to prevent it (Table 3).

The most commonly used source of information on management of UTIs was the internet (250, 63.8%). Both groups used the internet in comparable proportions (149, 61.3% vs. 101, 67.8%; *p* = 0.197). Internet use was followed by GPs (227, 57.9%), and again there were no significant differences between the two studied groups (134, 55.1% vs. 93, 62.4%; *p* = 0.157). The third most commonly used source of information was family and friends (192, 49.0%). A significantly larger proportion of non-biomedical students turned to friends and family compared to biomedical students (108, 44.4% vs. 84, 56.4%; *p* = 0.022). Pharmacists were a source of information for 148 (37.8%) women (83, 34.2% vs. 65, 43.6%; *p* = 0.061) and specialists for 65 (16.6%) women (38, 15.6% vs. 27, 18.1%; *p* = 0.522). Magazines were used by only 17 (4.3%) women (9, 3.7% vs. 8, 5.4%; *p* = 0.452).

Around three quarters of women (280, 71.4%) agreed with the statement that they could reach their GP in a timely manner should they experience symptoms of a UTI. As many as 146 (37.2%) had their urine tested when UTI symptoms occurred, 42 (10.7%) had tests repeated after treatment, 103 (26.3%) had tests only if the infection persisted and was not resolved after treatment, for 39 (9.9%) a test was performed only for the first episode, and 58 (14.8%) stated that it is was not necessary, leaving 4 (1%) who did not know. Around half of the women (201, 51.3%) would not consider management of UTI in pharmacy if it were available and 85 (21.7%) would consider it, while 31 (7.9%) would consider it if it were done in a private room in the pharmacy, 34 (8.7%) would consider it only if the infection was confirmed with a diagnostic test, and 39 (9.9%) would consider it only if the infection could by confirmed with a home diagnostic test. There were no differences between the two groups on this matter. Three quarters of women (292, 74.5%) were content with the working hours of pharmacies (*p* = 0.454). Half of women (218, 55.6%) always visited the same pharmacy, 20 (5.1%) mostly visited the same pharmacy, and 154 (39.3%) did not have a specific pharmacy they went to.

## 3. Discussion

In Croatia, health services are free of charge and women are free to choose their primary care physician [21,22]. Furthermore, antibiotics, except for certain topical powders and ointments, are prescription-only and impossible to obtain over-the-counter in pharmacies [23]. The results of this study show that most women feel they do not need an antibiotic for every UTI and that they have great confidence in recognizing a UTI. Prescription medications for prevention of UTIs were provided to 7.7% of women, while preventative antibiotics for recurrent UTI were prescribed to 22.2% of women. Upon presenting with a symptom of UTI, less than 5% reported self-medicating with antibiotics, while three-quarters waited, increased their fluid intake with or without use of painkillers, and contacted their GP if the symptoms did not resolve. The literature suggests that more than 55% of GPs believe that women can recognize symptoms of a UTI on their own [24]. In a recent Dutch study, around 50% of women diagnosed with UTI were considered knowledgeable on the subject. Greater knowledge was observed among younger women and women who had more than one UTI [25]. Most women in this study believed that they could reach a GP in a timely manner, while up to 30% would consider UTI management in a pharmacy and an additional 18.5% would consider it if it were possible to confirm an infection with an in-pharmacy or at-home test.

Several studies have revealed a decline in the amount of antibiotics prescribed for recurrent UTIs in younger women. The authors attribute this to an increased awareness of interstitial cystitis or bladder pain syndrome, which can present similarly to recurrent UTI but does not require treatment with antibiotics. A lower rate of dispensation of prophylactic antibiotics has been observed as well [26]. Furthermore, findings from recent research have suggested that a considerable proportion of women are willing to tolerate less than optimal management in certain respects in order to avoid antimicrobial treatment for UTIs. These results indicate that there is a preference to avoid antimicrobial treatment and that resistance is strongest among women with a higher level of education, while younger women or those who had only experienced one previous UTI placed greater emphasis on quicker resolution of complaints [27].

The results of the present research indicate that biomedical students have greater awareness of the risk of antibiotic resistance upon repeated exposure, have greater knowledge on possible interactions, and may be more open to the idea of delayed antibiotic prescription compared to non-biomedical students. According to published studies, women perceive antibiotics as a cure for UTI and find them to work quickly, while they perceive OTC treatments as more natural but less effective [28]. In a different study, 62% of women opted for non-antibiotic treatment for their most recent UTI, with 35% taking extra fluids, 27% taking cranberry juice, 21% taking pain-relieving medication, and 17% taking cystitis sachets. Women under 25 years of age more frequently took cranberry juice and looked up information online [5].

Overall, the women in this study reported use of cranberry preparations (54.8%), vitamin C (31.9%), D-mannose (24.7%), probiotics, and herbal preparations either for preventative purposes or during a UTI. We observed significant differences in the use of dietary supplements between the two studied groups, as greater use of dietary supplements for UTI was found in the group of non-biomedical students. There may be several reasons for this finding. According to a study conducted among women in the Netherlands, more than 80% of women experiencing symptoms of UTI used complementary and self-care strategies in addition to regular treatment, with most reporting use of cranberry (51.9%), vitamin C (43.8%), and D-mannose (32.7%), which were perceived as the most effective. Research has shown that many women experiencing recurrent UTIs would welcome alternative management to reduce their use of antimicrobials. Women gather information on self-care strategies for UTIs mostly from the internet, as just one-fifth of the women were informed on these strategies by their healthcare professional [16]. Furthermore, women reported that discussions about non-antibiotic treatments with healthcare practitioners were unusual and that OTC medications were presented as ineffective, though not as harmful [28]. Women with recurrent UTIs may lose trust in their health care provider and feel that they have not been acknowledged regarding previous treatments and their desires [17]. Moreover, non-biomedical students turned to family and friends for information significantly more often compared to biomedical students.

Furthermore, evidence and recommendations on the use of certain preparations are inconclusive. For example, women reported increasing fluid uptake on symptom onset, and they perceived cranberry juice as being superior to other fluids [28]. Although the exact mechanisms and efficacy of cranberry preparations for UTI remain unclear, studies have shown a decrease of up to 41% of in symptoms of UTI among women who drank cranberry beverage daily compared to those who drank a placebo [29]. Furthermore, research has shown that a combination of D-mannose with polyphenols or Lactobacillus may present another option for UTI prophylaxis. Among other possible treatments, D-mannose may be a promising nonantibiotic prevention strategy. D-mannose is excreted in urine, and inhibits bacterial adhesion to the urothelium. According to research, D-mannose may serve as a prophylaxis for UTI with fewer adverse effects compared to nitrofurantoin. Data confirm the potential of oral D-mannose for reducing of the risk of recurrent UTIs in women. D-mannose seems to be safe and effective as a non-antimicrobial prophylaxis for recurrent UTIs in women. However, the European Urological Guidelines on urinary tract infections do not recommend it for routine use [30,31].

Of the women in this study, 31.4% used oral probiotics and 23% stated that they used vaginal probiotics (mostly while having a UTI); overall, half believed that vaginal probiotics can either help reduce symptoms of infection or help prevent it. However, the research on this topic is inconclusive. For example, lower lactobacilli levels promote vaginal *E. coli* colonization, which increases the risk of UTI. This happens because vaginal lactobacilli inhibit colonization by potential pathogens by creating hydrogen peroxide and bacteriocins. The term “covert pathogenesis” refers to the phenomenon where, for example, *B. streptococcus* and *G. vaginalis* support the survival of *E. coli* in the bladder, promoting the development of UTIs. Furthermore, research indicates that the use of lactobacilli vaginal suppositories is linked to a considerable decrease in recurrent UTIs [32]. However, the results of systematic reviews of published data indicate that probiotics do not have a significant effect on the prophylaxis of UTIs in premenopausal women. Furthermore, it is not clear which interventional variables (i.e., routes of administration or bacterial strains) are the most effective [33].

A Dutch study revealed that around a third of women increased their fluid intake and searched the internet for information on self-management before consulting a GP for UTI symptoms, while 15% used analgesics and increased fluid intake. In the same study, 47% used cranberry, 5% used D-mannose, and 29% used vitamin C. Awareness of various preventive behavioral measures between 20% and 90% was reported [34]. Several behaviors are thought to increase the risk of recurrent UTIs, among which are reduced fluid intake, wiping from back to front after defecation, douching, wearing occlusive underwear, delaying urination, and post-coital urination as well as increased tone of the external sphincter during micturition [35]. Interestingly, significant differences were observed in the two groups on this matter as well, as biomedical students were significantly more likely to wipe front to back for prevention and management of UTIs, while the non-biomedical group chose cotton underwear during UTIs. While this may be due to personal preference, it could be influenced by the sources of information and by evidence for such claims (i.e., representation in guidelines for management of UTIs). Indeed, the women in this study were younger and more frequently turned to the internet for information (63.8%) compared to the aforementioned study, which included women of all ages, of whom around 40% used the internet as a source of information on UTIs [34], while GPs were a source for 57.8% and pharmacists for 37.8%. This suggests a need for greater engagement of health-care professionals in management and self-care of UTIs.

As this was a single-center study, the results of the present study may not be easily generalizable. It is highly unlikely, though not impossible, that a proportion of students with limited digital skills could have been excluded from the study. Another limitation is that this study could not investigate exact behaviors, as it was survey-based and self-reported. However, as the study was anonymous, we do not expect any bias regarding this. It is crucial for antibiotics to be observed regarding their spectrum and potential for increasing resistance. However, in this study we only questioned women anonymously, and had no insight into their medical data; thus, we do not have any data on which antibiotics were prescribed for prevention and treatment of UTIs. It is possible that women who had experienced a UTI were more likely to participate in this study.

## 4. Materials and Methods

This was a cross-sectional survey-based study conducted among female students at the University of Split in the academic year 2022/2023. After completing a literature search, we decided to use a previously published tool.

### 4.1. Survey

The tool by Lelie-van der Zande et al. [34] includes recommendations from the UTI guidelines of the Dutch College of General Practitioners. As the Interdisciplinary Section for Antibiotic Resistance Control guidelines on antimicrobial treatment and prophylaxis of urinary tract infections in the Croatian national guidelines are not fully aligned with the Dutch guidelines, and the studies were conducted among study populations with different ages, several adjustments had to be made to the original version of the tool. For this purpose, a pharmacologist, a pharmacist, and a general practitioner were consulted. First, we expanded the multiple-choice questions (MCQ) with answers that were frequently collected as “other” written by study participants in the study by Lelie-van der Zande et al. [34]. Second, we expanded the existing survey to include traditional herbal medicines, herbal medicines, over-the-counted medications, dietary supplements, and other treatments marketed and available in pharmacies in Croatia that were not included in the original version of the survey. A distinction was made between oral and vaginal probiotic formulations. Furthermore, the survey was expanded to include other possible options for preventing and easing symptoms of UTIs, such as avoiding restrictive clothing, opting for cotton underwear, and avoiding use of daily pads, as these were suggested by general practitioners as practices often seen among women. In addition, the survey collected information about visits to pharmacies and opinions on possible UTI management in pharmacies. Finally, the survey was expanded with six statements rated on a 5-point Likert scale to further investigate attitudes of female students related to the use of antibiotics and probiotics for UTIs. This, along with questions related to age and faculty, added up to 36 survey items.

The final version of the survey was tested among ten non-biomedical students for readability and length. Two minor language adjustments were made. The results from these students were not included in the analysis. The survey was then prepared as a Google Forms document and distributed as an online link via student representatives in different faculties.

### 4.2. Study Participants

According to the total number of 10,883 female students attending the University of Split in the academic year 2022/2023, with a confidence level set at 95% and a margin of error of 5%, a sample size of 372 students was calculated using the freely available online Sample Size Calculator tool from SurveyMonkey [36]. The study was approved by the Ethics Committee of the University of Split School of Medicine.

Participation in this study was voluntary and completely anonymous. The participating students received no compensation, and could withdraw at any point. A brief notice for participants was included at the beginning of the survey to the effect that completing the survey was deemed acceptance of participation in the study. Furthermore, the survey did not gather any data that could be traced back to identify the participants.

### 4.3. Statistical Analysis

All data were analyzed using descriptive statistics. The students were divided in two groups based on the school they attended: either a biomedical field (i.e., medicine, pharmacy science, nursing, etc.) or a non-biomedical field (i.e., law). Whole numbers and proportions are used where applicable in ordering biomedical vs. non-biomedical students. The chi square test or Fischer’s exact test was used to compare the differences between the two groups for categorical variables. Statistical significance was set at *p*-value < 0.05. All data were analyzed using MedCalc version 19.1.2. (MedCalc Software Ltd., Ostend, Belgium).

## 5. Conclusions

This study revealed that young women are confident in recognizing a UTI, are open to alternative treatments, and are willing to consider UTI management in a pharmacy setting. On the other hand, it reveals that there might be gaps in their knowledge regarding the risk of antibiotic resistance, possible interactions, and the efficacy of available preparations, as students from the group of biomedical schools showed greater knowledge and different behaviors with respect to these topics. Our results indicate a need for greater involvement of health-care practitioners in discussing UTIs with women.

## Figures and Tables

**Table 1:** Measures taken by women when they had an UTI.

	Measures Taken by Women While They Had an UTI				Measures Taken by Women to Prevent UTI			
	All	Biomedical	Non-Biomedical	*p*-Value *	All	Biomedical	Non-Biomedical	*p*-Value *
Increase fluid intake	334	209	125	0.567	317	199	118	0.510
	(85.2%)	(86.0%)	(83.9%)		(80.9%)	(81.9%)	(79.2%)	
Not postponing micturition	309	191	118	0.889	299	189	110	0.373
	(78.8%)	(78.6%)	(79.2%)		(76.3%)	(77.8%)	(73.8%)	
Using cotton underwear	250	143	107	0.010	251	157	94	0.761
	(63.8%)	(58.8%)	(71.8%)		(64.0%)	(64.6%)	(63.1%)	
Wiping buttocks from front to back	196	132	64	0.029	247	171	76	<0.001
	(50.0%)	(54.3%)	(43.0%)		(63.0%)	(70.4%)	(51.0%)	
Emptying bladder soon after sexual intercourse	178	119	59	0.071	219	144	75	0.085
	(45.4%)	(49.0%)	(39.6%)		(55.9%)	(59.3%)	(50.3%)	
Complete emptying of the bladder	219	139	80	0.497	163	106	57	0.296
	(55.9%)	(57.2%)	(53.7%)		(41.6%)	(43.6%)	(38.3%)	
Washing without (alkaline) soap	110	70	40	0.675	122	73	49	0.555
	(28.1%)	(28.8%)	(26.8%)		(31.1%)	(30.0%)	(32.9%)	
Avoid tight clothes	122	76	46	0.933	101	64	37	0.741
	(31.1%)	(31.1%)	(30.9%)		(25.8%)	(26.3%)	(24.8%)	
Avoid daily sanitary pads	117	59	58	0.002	146	93	53	0.592
	(29.8%)	(24.3%)	(38.9%)		(37.2%)	(38.3%)	(35.6%)	
Shower before sex	71	47	24	0.420	108	72	36	0.240
	(18.8%)	(19.3%)	(16.1%)		(27.6%)	(29.6%)	(24.2%)	

* chi-square test; data are presented as numbers (proportions).

**Table 2:** Preparations taken by women for treatment and prevention of UTIs.

	Taken Only during UTI			Taken Every Day for Prevention of UTI			
	All	Biomedical	Non-Biomedical	All	Biomedical	Non-Biomedical	*p*-Value *
Cranberry preparations	180	98	82	35	22	13	0.360
	(83.7%)	(54.4%)	(45.6%)	(16.3%)	(62.9%)	(37.1%)	
Vitamin C	47	27	20	78	42	36	0.696
	(37.6%)	(57.4%)	(42.6%)	(62.4%)	(53.8%)	(46.2%)	
Oral probiotics	76	49	27	47	26	21	0.314
	(61.8%)	(64.5%)	(35.5%)	(38.2%)	(55.3%)	(44.7%)	
D-mannose	81	46	35	16	13	3	0.068
	(83.5%)	(56.8%)	(43.2%)	(16.5%)	(81.2%)	(18.8%)	
Vaginal probiotics	78	43	35	12	9	3	0.197
	(86.7%)	(55.1%)	(44.9%)	(13.3%)	(75.0%)	(25.0%)	
Preparations of *uva ursi*	62	39	23	4	4	0	0.134
	(93.9%)	(62.9%)	(37.1%)	(6.1%)	(100%)	(0%)	
Herbal oral drops	48	28	20	5	5	0	0.070
	(90.6%)	(58.3%)	(41.7%)	(9.4%)	(100%)	(0%)	
Herbal tablets	46	29	17	5	5	0	0.099
	(90.2%)	(63.0%)	(37.0%)	(9.8%)	(100%)	(0%)	

* chi-square test or Fischer’s exact test; data are presented as numbers (proportions).

**Table 3:** Women who agreed or fully agreed with the following statements.

	All	Biomedical	Non-Biomedical	*p*-Value *
An uncomplicated infection can go away even if it is not treated with antibiotics	260	168	92	0.175
	(66.3%)	(69.1%)	(61.7%)	
Antibiotic treatment may reduce the effectiveness of repeated treatment with the same antibiotic	240	167	73	0.002
	(61.2%)	(68.7%)	(49.0%)	
Probiotics (vaginal) can help reduce the symptoms of infection	204	127	77	0.349
	(52.0%)	(52.2%)	(51.7%)	
Probiotics (vaginal) can help prevent re-infection	194	126	68	0.651
	(49.5%)	(51.9%)	(45.6%)	
Antibiotics can reduce the effectiveness of oral hormonal contraception	108	86	22	< 0.001
	(27.3%)	(35.4%)	(14.8%)	
It is safe to delay the use of antibiotics and start antibiotic treatment 3 days after the onset of symptoms	106	71	35	0.036
	(27.0%)	(29.2%)	(23.5%)	

* chi-square test; data are presented as numbers (proportions).

## Data Availability

The raw data are available from the corresponding author upon reasonable request.
